# Brief group-delivered motivational interviewing is equally effective as brief group-delivered cognitive-behavioral therapy at reducing alcohol use in risky college drinkers

**DOI:** 10.1371/journal.pone.0226271

**Published:** 2019-12-10

**Authors:** Cristina Martín-Pérez, Juan F. Navas, José C. Perales, Ángela López-Martín, Sergio Cordovilla-Guardia, Mónica Portillo, Antonio Maldonado, Raquel Vilar-López

**Affiliations:** 1 Mind, Brain and Behavior Research Centre, University of Granada, Granada, Spain; 2 Department of Experimental Psychology, Autonomous University of Madrid, Madrid, Spain; 3 Department of Experimental Psychology, University of Granada, Granada, Spain; 4 Biomedical Research Management Foundation, Cádiz, Spain; 5 Nursing Department, Nursing and Occupational Therapy College, University of Extremadura, Cáceres, Spain; 6 Mental Health Services, Ribera Hospital, Valencia, Spain; 7 Department of Personality, Evaluation and Psychological Treatment, University of Granada, Granada, Spain; University of the Witwatersrand, SOUTH AFRICA

## Abstract

College students are particularly vulnerable to risky alcohol use, which increases their likelihood of developing an alcohol use disorder in the future. As such, preventing and reducing alcohol use among college students should be a priority for health and social policies. This work was aimed to show that brief group-delivered MI is as effective as brief-group CBT at reducing alcohol use in college students. Eighty-nine college students (69 females; mean age = 21.01, SD = 2.85) with risky alcohol use, as measured by the AUDIT-C, were assigned to two groups, receiving three sessions of either brief group-delivered MI or CBT (bMI/bCBT). Alcohol use was assessed 3 and 6 months after the interventions, and analyzed according to an Intention-to-treat design. Changes in alcohol use at both points (relative to baseline) as well as post-intervention scores of intention to continue treatment and satisfaction with the psychologist were compared across groups, using one-sided Bayesian t-tests. Alcohol use decreased in both groups at the 3- and 6-months measurement points (relative to baseline). However, using bCBT superiority as an alternative hypothesis and the absence of such superiority as a point-null hypothesis, the Bayes factors supported the null at both the 3- and the 6-months follow-up (BF_01_ = 7.13, and BF_01_ = 5.22 respectively). Furthermore, the intention to continue treatment was substantially higher in the bMI group (BF10 = 9.77). These results are considerably robust to changes in analyses’ priors. This study suggests that bCBT is not more effective than bMI at reducing alcohol use in our college student group (in which females are overrepresented). Additionally, bMI showed higher intention to continue treatment scores. The comparable results of brief and group-delivered CBT and MI interventions in alcohol use reduction allows clinicians to select treatments based on their own skills or preferences without any detriment to efficacy.

## Introduction

Harmful use of alcohol has become a severe global health problem. Currently, 5.1% of the burden of diseases worldwide in individuals aged 15 or older is attributable to alcohol [1]. The health consequences of heavy alcohol use range from direct physical hazards (e.g. cardiovascular diseases, liver cancer) or neurobiopsychological conditions (e.g. major depressive disorder, anxiety disorders or substance use disorders) [2,3] to indirect ones such as injury or disability caused by violence or accidents during intoxication [4,5]. Additionally, 5.9% of global deaths in 2012 were due to alcohol and the consequences of heavy consumption [1].

Within the young adult age group, college students stand as a particularly vulnerable population to experiencing risky alcohol use [6,7] in comparison to their non-college counterparts [8,9]. This susceptibility, indeed, leads to an increased likelihood for exhibiting substance use-related problems and to developing an alcohol disorder in the future [7,10]. Thus, given the high prevalence of risky-drinking in college students [11], early interventions for the prevention and reduction of alcohol use in this population should be a priority for health and social policies [1]. Among the most commonly used strategies for addressing this problem, prevention programs aimed at reducing the amount of alcohol consumed is crucial with college students [12]. This particular approach is appropriate as it does not force them to an abstinence-only scenario and instead emphasizes the encouraging outcomes of alternative goals as controlled reduction in risky-drinking [13]. The task force of the National Institute on Alcohol Abuse and Alcoholism (NIAAA) has centered its focus on the efficacy of two evidence-based interventions for reducing alcohol use in college students: the Cognitive-Behavioral Therapy (CBT) and the Motivational Interviewing (MI) [6]. In addition to highlighting these types of intervention, this report as well as several studies [6,14,15] have emphasized the fact that brief interventions are preferable to traditional formats for college students.

CBT is the dominant paradigm for treating alcohol dependence and for tertiary prevention [16]. It is a directive and structured psychotherapy, encompassing a wide range of techniques aimed at changing maladaptive patterns of cognition, beliefs, and attitudes as determinants of behavior [17–19], that can be considered the gold standard for psychological interventions. Brief versions of CBT (between 1 and 5 sessions) have been implemented as a cost-effective alternative, and have also shown efficacy at decreasing alcohol use in young adults [15,20]. Contrary to alcohol dependence treatment, there is not a gold standard for reducing alcohol-use in secondary preventive interventions with college students. Nevertheless, brief MI has been used extensively in this population. MI stresses the dynamic nature of motivation and focuses on enhancing clients´ readiness to change by helping them explore and resolve their ambivalence regarding a potentially dangerous behavior (e.g. alcohol use; [21]). Despite being one of the most commonly used interventions on college students with risky alcohol use, the efficacy of bMIs in college students has been recently challenged in a meta-analytic study [22]. Further, bMIs are usually used as a mere component within a wider interventional program [23], thus making it difficult to know the specific benefits related to this kind of technique. Indeed, it is common to find diverse components of both CBT and MI mixed into the same intervention program for college students, such as the well-known BASICS or ASTP programs [24,25], commonly implemented on University settings. Nonetheless, a recent meta-analysis showed no statistically significant effects for the combination of these components in young adults [15].

To the best of our knowledge, there is no study comparing the brief versions of CBT and MI in college students with risky drinking behavior. To date, only one meta-analysis has shown a slightly better performance of the MI interventions over CBT in young adults with risk-drinking. However, this study was not a direct comparison of CBT and MI, and was not focused on college students [26]. Besides these statistical differences, other studies have emphasized the clinical relevance of brief MI, which have shown better treatment outcomes related to the client-centered style of MI. These improved outcomes include a better alliance and engagement to treatment (e.g. a greater attendance of clinical sessions), enhanced rapport with the therapist, and an increased intention to change alcohol use [21,27]. As long as MI can be regarded as equally effective as CBT in alcohol reduction effectiveness, these complementary features may render MI globally advantageous.

Finally, the best format for delivering the prevention interventions (i.e. individual vs. group-based) in college students is a matter of ongoing debate. A recent meta-analysis [15] showed similar outcomes for group and individual-delivered brief interventions in young adults. However, due to the importance of peer influence and feedback, clinicians and researchers have highlighted the clinical relevance of group interventions, especially in young adults [28,29]. Whereas there is extensive research on group CBT, there is very little on brief group-delivered MI. Furthermore, the results of existing studies are mixed and the founders of this therapy have suggested this issue to be an important area of interest for research [21].

Despite the literature briefly described above, the non-superiority of group-delivered brief Cognitive Behavioral Therapy (bCBT) relative to brief Motivational Interview (bMI) in the reduction of alcohol use in college students with risky alcohol use remains untested. The present study aims to explore the potential differences between both early interventions at producing different degrees of the intention to engage in future alcohol reduction sessions [30] or differences in satisfaction with the psychologist. Based on the previous literature, our main hypothesis is that the group-delivered brief MI intervention is as effective as group-delivered brief CBT at reducing alcohol use. Further, considering MI seems to produce higher acceptance, and enhanced perceived quality and satisfaction with the services as compared to other interventions [31], we hypothesize that MI will lead to a greater intention to continue intervention and an improved satisfaction with the psychologist.

## Materials and methods

### Participants and procedure

A convenience sample of 1,008 potential participants from several schools at the University of Granada was initially screened. In this screening, we informed participants that we were conducting a research project about the patterns of alcohol use among college students, and that according to their responses they may be contacted for posterior phases of the study. They were informed during screening that the additional phases would consist of several sessions related to their alcohol use. Participants were eligible if they agreed to give us their contact details for the posterior phases, demonstrated risky alcohol use (as indicated by an AUDIT-C score of 5 or higher for males and 4 or higher for females; [32]), and did not present any neurological or psychiatric disorders. A group of 567 (56.25%) undergraduates met this selection criterion. Eligible candidates were telephonically contacted in rounds of approximately 30 participants per day (the amount of calls a researcher was able to do in a working session) and were invited to participate in both the initial assessment and the following clinical sessions. Researchers explained that the study aimed to better understand how to help people with different patterns of alcohol use to reduce their consumption. Further, they informed potential participants that the study consisted of 3 sessions in which there were group discussions about risk factors, and possible current and future consequences of their alcohol use. Researchers stressed the fact that participants could attend as many of the three sessions as they liked. We randomly called 354 participants of whom 213 answered the phone, and 90 participants agreed to participate (42.25% of those who were initially contacted). One participant was excluded due to a previously diagnosed psychopathology, as assessed by an initial semi-structured interview performed by psychologists with at least a Master’s degree. The final sample, comprising 89 students (8.83% of the participants initially screened; mean age = 21.01 (2.85); 77.5% female), was informed once again about in the first evaluation session about the study’s objectives and progression. During this session, participants were able to raise any doubts or concerns, and signed a written informed consent. No statistical differences were found in age (*p* = .292), education (*p* = .272), sex (*p* = .767) and AUDIT (*p* = .378) scores between those who were screened and those who participated in the intervention. Participants did not receive any monetary compensation or benefit for their participation.

The present study is a viability study for a clinical trial pre-registered at ClinicalTrials.gov (NCT02159391), which originally set out to test the efficacy of MI for patients hospitalized after alcohol-related accidents. Due to difficulties with recruitment, we designed the present viability and preliminary study to test the adequacy of the assessment and intervention protocols. Further, we tested the results of group-delivered brief MI. The present study received approval from the Ethics Committee at the University of Granada (26/07/16/226).

**Fig 1:**
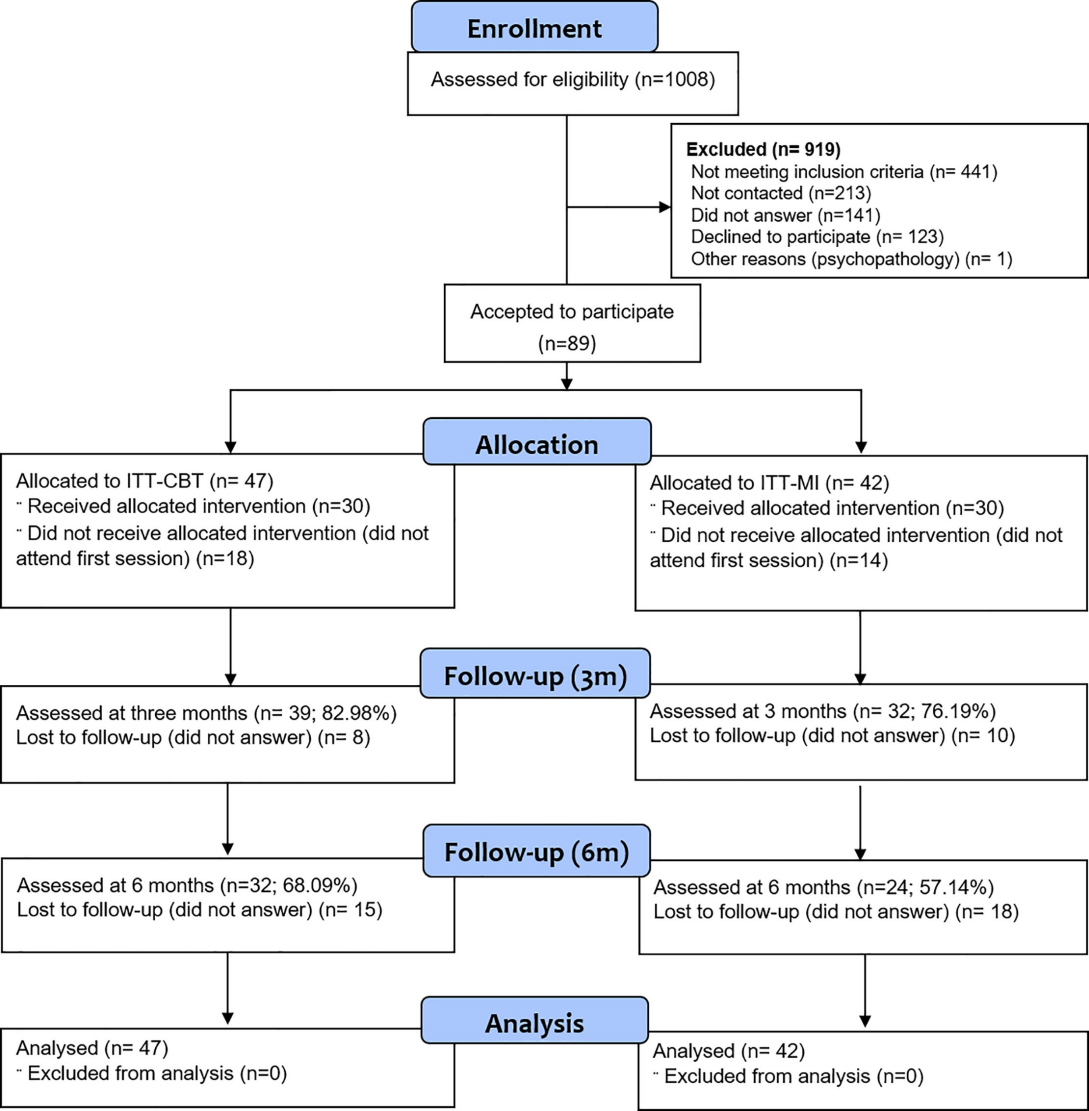
Flow Diagram of the two groups throughout the different study phases.

In the initial session, each participant was individually assessed with a comprehensive battery of cognitive abilities, personality, and alcohol-related measures (for more detailed information on the instruments associated with the aims of the study see ‘Measures’ below). After the initial assessment session, the PI of the project assigned students to groups, following their order of evaluation (participants 1 to 4 were assigned to one treatment, participants 5–8 to another treatment, and so on). Thus, the present study was not a randomized trial and there was no concealment of allocation. In sum, 47 participants (52.80% of the final sample) made up the bCBT intervention group, and 42 (47.19%) the bMI group (see Fig 1). Both interventions comprised three 1h 30’-long sessions that were administered in groups of 4 to 8 participants. After the last session, participants completed two tests: SOCRATES 8A and a Visual Analogue Scale about intervention outcomes (see ‘Measures’ below). It is important to note that both types of interventions were conducted by the same psychologist with four years of experience in psychological treatment. CBT interventions were carried out under the supervision of the sixth author and MI by the fifth author, who checked for fidelity to treatments and ensured that all of the active ingredients in both therapies were correctly applied. This was done to maximize internal consistency and to eliminate differences between therapies due to the psychologists’ individual characteristics. Further, the PI of the project assigned students to groups such that researchers performing the initial evaluation and assessing the outcomes were blind to the participants’ allocation. The psychologist was not involved in recruitment, in the initial, nor in the post-intervention assessment sessions.

Lastly, telephone follow-up interviews were carried out at 3 and 6 months after the initial assessment in order to explore the amount of alcohol participants had consumed in the preceding month. These interviews included the whole sample [including (i) non-compliers, namely participants who did not attend some of the proposed sessions for justified reasons, but later continued to be involved, and (ii) drop-outs, namely, those who abandoned the study]. In other words, partial or total non-attendance did not imply either being taken out of the sample for follow-up, or being excluded from further analyses. Still, some individuals were unreachable or decided not to participate in follow-up interviews. As such, 71 (79.8%) individuals were assessed at 3 months, and 56 (63%) at six months. Some participants were interviewed at 3 months but not at 6 months (n = 22), and vice versa (n = 7). Therefore, the samples for the two measurement points do not completely overlap, and analyses will be performed for both measurement points separately. Participants received a brief report of their neuropsychological profile including their performance in the initial assessment. All sessions regarding intervention and assessment were carried out in the research labs of the Mind, Brain and Behavior Research Center (CIMCYC) at the University of Granada.

### Measures

#### Alcohol use severity

The shortened version of the Alcohol Use Disorders Identification Test (AUDIT-C) is a brief validated screening test for risky alcohol use [33]. It consists of 3 items assessing frequency and amount of alcohol use, resulting in a total score ranging from 0 to 12. A cutoff score of 5 for males and 4 for females are the suggested cut-off points for detecting risky alcohol use in Spanish college students [32].

#### Outcome variables

*Recent alcohol use*. The Alcohol Timeline Followback (TLFB; [34]) has been used to measure total quantity of alcohol use. This assessment was completed at baseline and after 3 and 6 months. Using a calendar, individuals provide an estimation of their consumption within the past 30 days. The Alcohol TLFB demonstrated adequate psychometric properties when administered on-site and over the telephone, and in several drinker groups [34,35]. To administer the TLFB over the telephone, we asked participants to have their mobile phones and agendas on hand, along with a calendar. The evaluator took note of their daily alcohol use during the past 30 days in order to calculate the total alcoholic units consumed.

*Readiness to change*. SOCRATES 8A [36] measures the motivation to change in individuals with alcohol use. It is a 19-item questionnaire divided in three subscales: Recognition, Ambivalence, and Taking Steps. This questionnaire has shown sound psychometric properties in young adults [37].

*Motivation to continue treatment and satisfaction with the psychologist*. Two separated Visual Analogue Scales (VAS), ranging from 0–100, were administered immediately after the last treatment session in order to measure (i) their intention to engage in future alcohol-related interventions (“*Given the chance*, *to what extent would you be willing to attend to another session*?”, where 0 means “*I would never attend another session”* and 100 *“I would definitively attend another session”*), and (ii) the participants’ satisfaction with the psychologist (“*How satisfied do you feel about the time spent with the psychologist*?”, where 0 means *“I don’t feel satisfied at all”* and 100 *“I feel absolutely satisfied”*).

#### Interventions

*General common characteristics*. The interventions were group-delivered and administered in a brief format. Also, both interventions were administered by the same psychologist, which means that common abilities in psychotherapy (e.g. warmth, congruence, etc.) beyond specific intervention techniques that may influence therapeutic alliance [38] and therapy outcomes [39] were thereby matched for bMI and bCBT.

After the first and the second session, all participants were encouraged to perform homework tasks. These tasks are an active ingredient of CBT (e.g. [40]), as they are aimed at maintaining the new skills developed in the sessions. In order to control the time and effort invested in sessions for both interventions, homework tasks were also included in bMI groups with adapted content (see “motivational interviewing” below). For detailed information on both therapies, see S1 Appendix.

*Brief Cognitive Behavioral Therapy*. CBT is a directive and structured intervention, aimed at changing maladaptive thinking and behavioral styles. The intervention applied in this study was based on manuals for group-delivered CBT [41] and common applications of CBT in addiction and risky alcohol use [42,43].

All sessions were divided into different parts with a pre-established content. Each of the three sessions included a key technique of CBT interventions for risky alcohol use (i.e. [i psychoeducation, [ii] stimuli control, [iii] cognitive restructuring of alcohol-related cognitions and general automatic-irrational thoughts, and [iv] relapse prevention).

The aim of the first session was threefold. First, to implement a stimuli control technique, that is, to prevent or limit exposure to contextual stimuli (e.g. pubs, alcohol stores) that were previously linked to alcohol consumption and could boost alcohol intake [44]. Second, to identify alcohol-related cognitions, which refers to beliefs about the effects that alcohol may have over behavior (i.e. “*alcohol helps me to get along with peers*”); attitudes, that is, affective evaluations on alcohol; and motives, namely, the purposes driving alcohol consumption (i.e. “*I drink to have fun*”) [45]. And third, to identify general automatic-irrational thoughts that may be amplified by alcohol consumption (e.g. overgeneralization, selective attention, arbitrary inference).

The second session was aimed at cognitive restructuring of alcohol-related cognitions and general automatic-irrational thoughts by means of Socratic dialogue about their evidence, probability and utility. Further, it was achieved by promoting alternative ways of thinking regarding the effects of alcohol, attitudes, and motives.

The aim of the third session was to prevent relapse or an increase in consumption. Due to the fact that participants were encouraged to freely choose between quitting of reducing their alcohol use, different objectives were established depending on this choice during this session. In the case of those who chose to quit, the aim was to prevent any alcohol intake. As for participants who chose to reduce alcohol consumption, the aim was to prevent consuming an alcohol intake higher than the amount they desired to hold constant (i.e. three drinks per week). In addition, each session included a psychoeducational component, which consist of providing information about the negative physical (first session), psychological (second session), and social consequences (third session) of risky alcohol use. Doubts and myths about alcohol in relation to these consequences were clarified, and participants were offered a general overview about how to handle alcohol consumption.

*Brief motivational interviewing*. The MI applied in the present study followed the FRAMES model (feedback, responsibility, advice, menu of options, empathy, self-efficacy) [21] and previous clinical trials [31] following typical interventions in alcohol misuse. The main MI techniques applied were (i) open questions, (ii) reflective listening (iii) affirmations and (iv) summaries (see S1 appendix for a more detailed explanation of these techniques).

MI is a semi-directive and goal-oriented intervention used to explore and resolve patients’ ambivalence about alcohol use. Ambivalence could be defined as a complex pattern of opposing feelings and thoughts regarding the positive and negative consequences of maintaining certain behaviors [21]. All sessions were semi-structured and were designed to help participants resolve their ambivalence and to promote ambivalence in those with a low awareness of risky alcohol use. To do so, the psychologist promoted verbal expression of feelings and thoughts in a collaborative conversation in order to help them discover their own motivation to change, and thus facilitate commitment. This active collaborative dialogue was performed with an underlying attitude of acceptance, compassion and evocation (instead of correction).

Most participants expressed their desire to reduce or quit their alcohol use at some moment during the three sessions, although it is worth noting that participants chose the aims of the intervention (aims were not given by the psychologist). Participants were assigned homework at the end of the first and second sessions. These tasks consisted of writing the pros and cons of maintaining their current alcohol use (decisional balance) and two stories about their hypothetical future lives with and without alcohol consumption.

### Statistical analysis

An intention-to-treat (ITT) between-participant protocol was used for this study (Table 1). Groups consisted of 44 individuals in the ITT-bMI group, and 48 in the ITT-bCBT group. The ITT groups not only included participants who effectively received the planned treatment, but also those selected for treatment who did not attend, or only partially attended. This was done with the aim of preventing some of the common problems in randomized trials, such as effect inflation attributable to selectively differential non-compliance [46,47].

**Table 1:** Demographics and clinical characteristics of the study groups.

	Baseline				Baseline (participants with follow-up at month 3)				Baseline (participants with follow-up at month 6)			
	ITT-bMI (N = 42) M(SD)	ITT-CBT (N = 47) M(SD)	Stat	*p*/*BF_10_	ITT-bMI (N = 32) M(SD)	ITT-CBT (N = 39) M(SD)	Stat	*p*/*BF_10_	ITT-bMI (N = 24) M(SD)	ITT-CBT (N = 32) M(SD)	Stat	*p*/*BF_10_
**Age**	22.02 (2.71)	20.1 (2.7)	-3.347	.001/ 25.48	21.56 (2.36)	19.79 (1.85)	-3.541	.001/40.50	21.63 (2.36)	19.91 (1.99	-2.956	.005/8.84
**Sex (females)**	33 (78.6%)	36 (76.6%)	0.050	0.824	27 (84.4%)	30 (76.9%)	0.617	0.43	19 (79.2%)	25 (78.1%)	0.009	.925
**Education (years)**	14.69 (1.85)	14.85 (2.60)	0.331	.741/ 0.23	14.78 (1.81)	14.85 (2.1)	0.138	.891/0.25	14.42 (1.72)	14.84 (2.20)	0.787	.435/0.35
**AUDIT-C (total)**	6.19 (1.59)	6.77 (2.16)	1.439	.154/0.61	6.19 (1.69)	6.44 (1.97)	0.563	.576/0.30	5.96 (1.60)	6.59 (2.28)	1.164	.250/0.48
Scores 4–7	26 (61.9%)	24 (51.1%)			20 (62.5%)	22 (56.4%)			17 (70.8%)	17 (53.1%)		
Scores >7	16 (38.1%)	23 (48.9%)	1.059	.303	12 (37.5%)	17 (43.6%)	0.270	.603	7 (29.2%)	15 (46.9%)	1.803	.179
**SOCRATES** REC	10.48 (4.32)	11.89 (4.82)	1.455	.149/0.56	11.28 (4.64)	11.97 (5.04)	0.552	.597/0.29	11.0 (3.86)	11.44 (3.88)	0.419	.677/0.29
AMB	7.36 (3.88)	8.62 (3.52)	1.607	.112/0.68	8.22 (4.03)	8.46 (3.66)	0.266	.791/0.25	8.25 (4.02)	8.59 (3.22)	0.355	.724/0.29
STEP	15.81 (7.36)	15.79 (5.96)	-0.016	.988/0.22	16.59 (7.42)	15.46 (6.30)	-0.695	.489/0.30	17.04 (7.38)	15.13 (5.59)	-1.064	.294/0.45

*Note*: Between-participants’ differences were evaluated with independent samples t-tests in all cases, except for sex and AUDIT-C where chi-square tests were used. Abbreviations: ITT-bMI/bCBT = intention-to-treat of brief Motivational Interviewing/Cognitive-Behavioral Therapy; Stat = Statistics; p = p-value

*BF_10_ = Bayes Factor (for continuous variables)

M(SD) = mean (standard deviation)

REC = recognition; AMB = ambivalence.

Considering we were interested in interpreting null results, we analyzed data within a Bayesian framework [48]. A Bayes Factor (BF) in Bayesian inference, expressed as a ratio, is the relative support the observed data provides for each of two possible models (one representing the null hypothesis and the other the alternative hypothesis). Given a number of prior assumptions [49], a Bayes factor expresses the degree to which we should change our previous relative belief in the alternative hypothesis versus the null (or the null versus the alternative) in view of the collected evidence. Bayes factors thus yield three possible results: data supporting the alternative hypothesis model, data supporting the null hypothesis model, or inconclusive results. Additionally, although in BF analyses large samples are more likely to yield substantial evidence (in favor of either the null or the alternative), BFs are interpretable regardless of sample size.

First, in order to ensure ITT-bMI and ITT-bCBT groups did not differ at baseline, we performed two-sided Bayesian t-tests on TLFB scores. Two separated two-sided Bayesian t-tests were performed for individuals who were later assessed at 3 and 6 months.

Secondly, we performed two Bayesian JZS ANOVAs with JASP default settings to test pre-post effects on TLFB scores, from baseline to the 3-month and 6-month follow-up assessments.

Third, our main prediction was that bMI is as effective as bCBT at reducing alcohol consumption. Or, in other words, we were seeking support for the null (H_0_) against the directional alternative hypothesis (H_1_) that the CBT would be superior to MI. In the implementation of BFs used here, the null model is represented by a point-null hypothesis (the effect of interest is zero, such that the two treatments are equally effective in populational terms). The alternative model, on the other hand, is a composite hypothesis in which the median expected effect and its standard deviation are determined by selecting a prior distribution (a zero-truncated Cauchy distribution with a 0.707 width as the default, and one-sided JASP Bayesian t-tests to test the potential superiority of bCBT). Following the most common interpretation [50], substantial support for the null would be reflected by a Bayes Factor (BF_01_) larger than 3 (or what amounts to be the same, a BF_10_<0.33; for the qualitative interpretation of BF values).

A secondary prediction established that MI would yield a greater intention to continue treatment and more satisfaction with the psychologist than CBT. To test this, we performed one-sided Bayesian t-tests on these measures with MI superiority as the alternative hypothesis. In this case, substantial support for the alternative hypothesis would be reflected by a Bayes Factor (BF_10_) larger than 3. All analyses were performed in JASP 0.8.3.1 [51,52].

In addition to the analyses above, we performed a complementary per-protocol analysis to show the comparison between groups in participants who attended interventions and those who did not. These results are detailed in S2 Appendix.

## Results

Detailed information about the socio-demographic data and clinical measures are described in Table 1. Differences are reported for comparisons between ITT-bCBT and ITT-bMI participants who were assessed at 3 months, and for those who were assessed at 6 months. Importantly, in both cases, the two treatment groups differed in age (with participants receiving bCBT were younger than those receiving bMI). Analyses were conducted to discard the possibility that this difference affected results in the main analyses.

### Baseline differences

For participants who were assessed at 3 months, mean (SD) baseline TLFB scores were 39.04 (32.91) and 40.45 (23.68) for the ITT-bCBT and ITT-bMI groups, respectively. The *t*-test yielded a Bayes factor that substantially supported the null hypothesis (BF_10_ = 0.25); that is, supporting the absence of baseline differences between the two groups. Similarly, for participants who were assessed at 6 months, mean (SD) baseline TLFB scores were 34.71 (27.05) and 35.62 (19.66) for the ITT-bCBT and ITT-bMI groups, respectively. Again, the Bayes factor substantially supported the null hypothesis (BF_10_ = 0.27).

### Pre-post differences

Mean (SD) TLFB scores at 3 months were 27.95 (28.26) and 23.65 (20.18) for participants in the ITT-bCBT group and the ITT-bMI group (baseline means are reported in the previous subsection). The ITT group (ITT-bCBT, ITT-bMI) x pre-post (Baseline, 3 months) ANOVA yielded a BF_10_ = 589.82 for the pre-post effect (comparing the pre-post model against the null, no-effect model). Data anecdotally supported the absence of a group x pre-post interaction effect (BF_10_ = 0.34, computed from comparing the saturated model, H_1_, against the equivalent one without the interaction term, H_0_).

**Fig 2:**
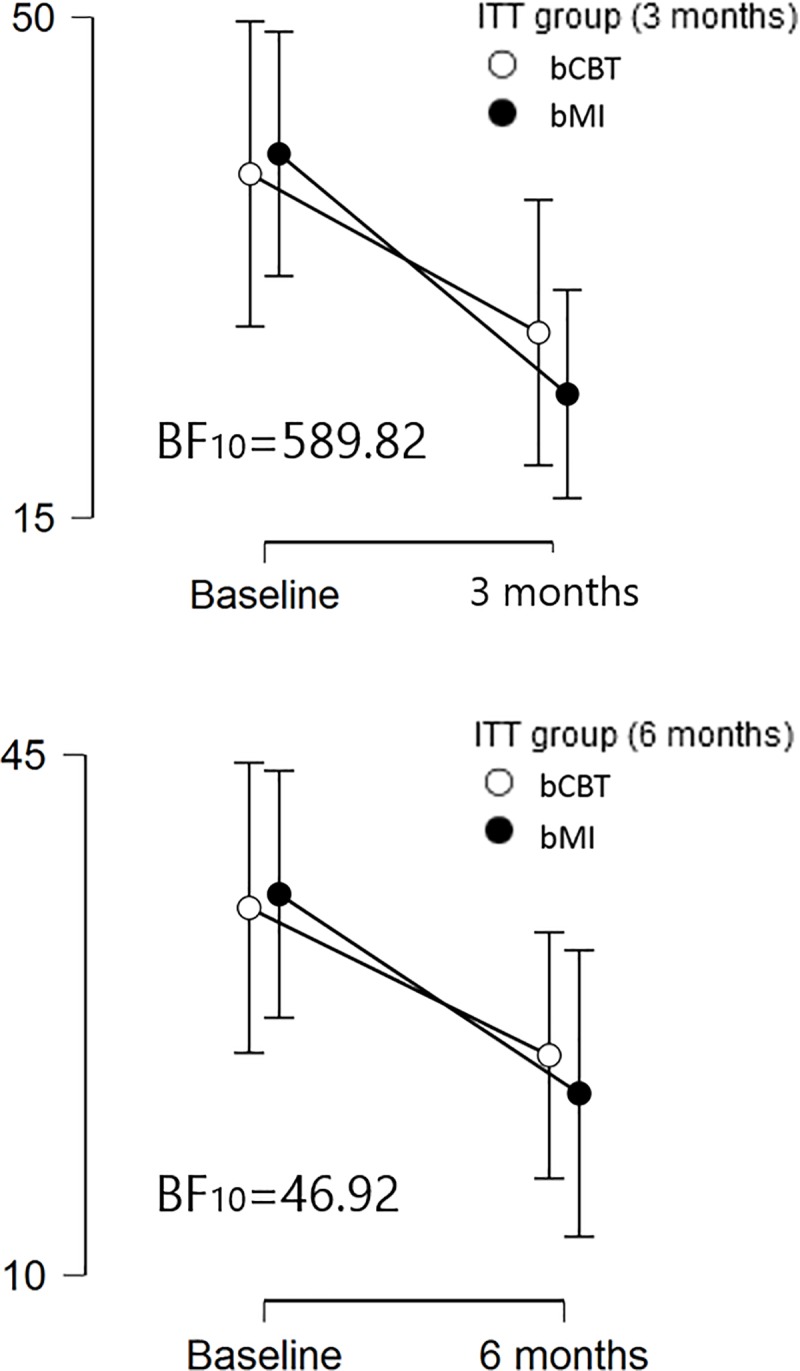
Differences between ITT-bCBT and ITT-bMI groups. Differences in alcohol use between: A) Baseline assessment and the 3-month follow-up and B) Baseline and the 6-month follow-up. Abbreviations: ITT, Intention-To-Treat; bCBT, brief Cognitive-Behavioral Therapy; bMI, brief Motivational Interviewing; TLFB, Timeline FollowBack, BF_10_, Bayes Factor.

Similarly, mean (SD) TLFB scores at 6 months were 24.78 (22.95) and 22.23 (22.79) for participants in the ITT-bCBT group and the ITT-bMI group. The ITT group (ITT-bCBT, ITT-bMI) x pre-post (Baseline, 6 months) ANOVA yielded a BF_10_ = 46.92 for the pre-post effect (comparing the pre-post model against the null, no-effect model). Data substantially supported the absence of a group x pre-post interaction (BF_10_ = 0.30). The pre-post differences in TLFB scores in both ITT-bCBT and ITT-bMI are displayed in Fig 2.

### Per protocol analysis

Complementary analyses (see S2 Appendix) were performed to test the degree to which actually receiving the intended treatment influenced alcohol consumption. Treatment (actual bCBT, actual bMI, no treatment) was decomposed into two dummy variables, each variable contrasting each of the treatments against the other two conditions. At 6 months, the reduction of alcohol use was larger for the bMI treatment than for the other two conditions, whereas there was no superiority of actual bCBT over actual bMI and no treatment when considered together.

### Equivalence Bayesian tests

We used one-sided Bayesian t-tests to assess our main hypothesis that the bMI intervention would be equally effective as bCBT. The output variable was the change observed in TLFB scores from baseline to the 3-month or 6-month assessment points. The Bayes factors will be expressed here as BF_01_, where *bMI is inferior to bCBT* (or bCBT is superior to bMI) as H_1_, and *bMI is as effective as bCBT* as H_0_.

Mean TLFB changes from baseline to the 3-month assessment were -11.09 (27.54) and -16.08 (24.09) for the ITT-bCBT and ITT-bMI groups, respectively, such that the decrease in alcohol use was visually larger for the ITT-bMI. In accordance with our hypothesis, the BF_01_ was 7.13 (credible interval of the effect size, CI: [-0.36, -0.003], *median* = -0.095), substantially supporting the model of bCBT non-superiority. Similarly, mean TLFB changes from baseline to the 6-month assessment were -9.92 (19.90) and -13.39 (27.82) for the ITT-bCBT and ITT-bMI groups, respectively, BF_01_ = 5.22 (credible interval of the effect size, CI: [-0.45, -0.005], *median* = -0.12). These findings support the hypothesis of bCBT non-superiority. Results are displayed in Fig 2.

We performed two linear regression analyses to check whether age differences between treatment groups exerted any effects on TLFB alcohol use reduction. In the first regression, for participants assessed at three months, ITT-group and age entered the analysis as predictors of TLFB alcohol use reduction (at 3 months). None of the two variables showed a significant effect. However, for participants assessed at 6 months, the age predictor showed a significant impact on alcohol use reduction (Age *β* = 0.499, *t* = 3.816; *p* < .001; bCBT vs. bMI *β* = -0.260; *t* = -1.99; *p* = .052).

With this in mind, we used the unstandardized regression coefficient for age to correct alcohol reduction scores in participants assessed at 6 months. Differential TLFB individual scores (*dif TLFB*_*i*_) were corrected as *dif TLFB*_*i*_—*B*_*age*_ · (*age*_*i*_−mean (*age*)), where *dif TLFB*_*i*_ is the alcohol use reduction for participant *i*, *B*_*age*_ is the unstandardized regression parameter of age over alcohol use reduction, *age*_*i*_ is the participant’s age, and mean (*age*) is the averaged age for all participants assessed at 6 months [53].

The main Bayesian test for these participants was re-run with corrected scores. Results show that the age correction did not qualitatively alter the implications of original results. The bMI group did not show less reduction of alcohol than the bCBT group. In fact, support for the null is even more convincing (the BF transitioned from substantially to strongly supporting the null after correction, BF_01_ = 10.35).

Given that BFs are very sensitive to the selection of priors, we performed robustness checks for main bMI non-inferiority Bayesian tests at 3 and 6 months. Qualitative conclusions (BF_01_ falling below 3) changed with the prior width corresponding to a small-very small effect in the 3-month analysis, and with the prior width corresponding to a small effect in the 6-month analysis (see S3 Appendix). In other words, our analyses substantially or strongly support H0 against a range of composite H1s, with median effect sizes ranging from medium to large. This conclusion converges with credible interval values, which are more robust than BFs to changes in prior selection [54].

### Intention to continue treatment and participants’ satisfaction with the psychologist

Our secondary hypothesis was that bMI would elicit more intention to attend future alcohol treatments and more subjective satisfaction with the psychologist. We performed a one-sided Bayesian t-test to explore the hypothesis that participants in the MI group would show a greater intention to continue with treatment after the last session. Bayes factor, expressed as BF_10_, with *MI triggers more intention to continue treatment than CBT* as H_1_.

**Fig 3:**
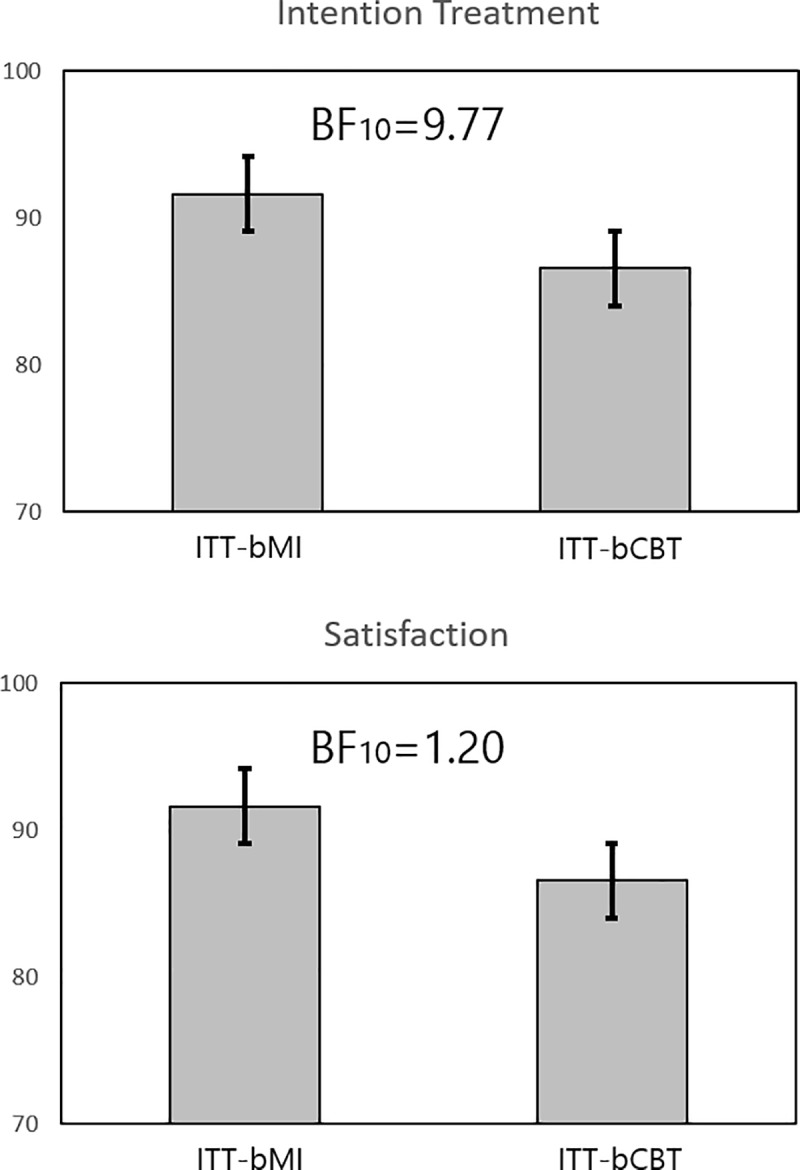
**Between-group differences in A) satisfaction with the psychologist and B) Intention to continue treatment.** Abbreviations: ITT, Intention-To-Treat; bCBT, brief Cognitive-Behavioral Therapy; bMI, brief Motivational Interviewing; BF_10_, Bayes Factor.

Mean intention-to-continue scores for the 50 participants who completed the post-intervention assessment were 78.96 (17.86) and 91.30 (13.33) for the bCBT and bMI groups, respectively, which yielded a BF_10_ = 9.77. This finding supports the prediction of greater willingness to continue treatment for the MI group. Furthermore, mean scores of satisfaction with the psychologist were 86.56 (11.42) and 91.61 (12.83) respectively for bCBT and bMI. The BF_10_ = 1.20 did not yield substantial support for the null nor the alternative hypothesis. The differences between ITT-bMI and ITT-bCBT groups in intention to continue treatment and participants’ satisfaction with the psychologist are shown in Fig 3.

## Discussion

To our knowledge, this is the first time group-delivered brief Motivational Interview (bMI) and group-delivered brief Cognitive Behavioral Therapy (bCBT) have been compared in terms of the reduction of alcohol use in college students with risky alcohol use. Our results support the hypothesis that group-delivered brief MI is as effective as CBT at reducing alcohol use in college students.

Preliminary analyses showed consumption decrements in both groups, as measured by differential TLFB scores, at the 3- and 6-month follow-ups. Although these decrements must be interpreted with caution in the absence of a proper untreated control group, they align with the previously reported effectiveness of group-delivered interventions [55] and brief administration formats in young adults [15].

Although CBT is the predominant intervention approach in addiction treatment and tertiary prevention [16], it does not seem to be the gold standard in the secondary prevention of risky alcohol use among college students. Our results suggest that MI is equally effective as CBT at reducing alcohol use in college students. Overall, this supports previous findings in young adults showing that MI and CBT produce similar outcomes regarding the reduction of alcohol use [15]. A complementary aim of our study explored whether intention to continue treatment (when the corresponding intervention had finished) and satisfaction with the psychologist would be higher in bMI than in bCBT. In this regard, bMI showed a better outcome in intention to continue treatment than bCBT, in line with other studies with non-seeking treatment individuals, although this time with alcohol use disorders and in mandated college students [55,56]. The difference in intention to continue treatment is relevant because it is likely to reflect the increase in problem awareness attributable to interventions. Also, it reinforces MI as a useful strategy to involve college students in treatment and prevention, thus surpassing the tendency of young individuals to defy adult control [57]. Contrary to our hypothesis, however, participants in the bMI group were not substantially more satisfied with their psychologist. In this case, the BF showed that the two hypotheses (superiority and non-superiority) were equally credible, such that no clear conclusions can be drawn from the null result. Nevertheless, this finding supports that MI is associated with a higher intention to continue treatment that is not attributable to the personal characteristics of the psychologist.

Per protocol complementary analyses to test the degree to which actually receiving the intended treatment influenced alcohol consumption showed that receiving treatment did make a difference, but only if that treatment was the bMI.

The novelty of our study relies on two features. First, as far as we know, this is the first study to compare brief, group-delivered versions of CBT and MI interventions. Furthermore, our sample consisted of non-pathological (but in-risk) college students with a risky alcohol use, who were not seeking treatment, and thus had low risk awareness. Indeed, according to clinical observations, the great majority of our participants were pre-contemplative or contemplative, and until they began the interventions, almost none of them had regarded their alcohol use as potentially risky.

Our results should be considered in the context of some limitations. First, the study was not a randomized controlled trial, and there was no control condition. Further, we used a convenience sample of college students, in which females were overrepresented, although we performed an extensive screening in several areas to increase the heterogeneity of the sample. Moreover, the sample size may limit the generalizability of our data. This problem combined with drop-outs led to a decreased sample size when making comparisons. Bayesian analyses allowed us to partially surpass this problem, as sufficiently high or low BFs are interpretable regardless of sample size. In other words, although small samples are less likely to yield sufficiently large BFs in any direction, if they do, these can be interpreted using the customary standards. Finally, the same psychologist applied both interventions and this could compromise the generalizability of our findings. However, this study characteristic made it possible to control for the influence of the psychologist’s experience, personality or therapeutic skills, and its influence over clinical outcomes regardless of the psychological intervention [38,58]. Lastly, we cannot rule out that assessment reactivity may have contributed to the reported reductions in alcohol use [59,60], especially considering that participants received feedback on their neuropsychological performance, and this could serve as an active ingredient prompting change across conditions. Nevertheless, none of the participants showed any neuropsychological deficits, and thus the possible effect of this feedback on the results should have been very limited.

In sum, the strength in our study is rooted in our novel approach, including brief and group-delivered versions of empirical-based interventions. In practical terms, our findings have potential applicability in public social and health systems. The introduction of these group-delivered brief interventions in primary care, high schools and other similar institutions, may help reduce alcohol use. In turn, the rates of future substance use-related problems may also decrease, which could significantly diminish the costs for public policies regarding alcohol use problems [61]. Furthermore, the comparable results of brief and group-delivered CBT and MI interventions in alcohol use reduction could be helpful for clinicians when selecting one or the other based on their own skills or preferences without any detriment to efficacy. Nonetheless, professionals should keep in mind that MI may provide an extra advantage in boosting college students’ intention to follow extended treatments. Finally, it is worth noting that MI, unlike CBT, was designed to be implemented not only by psychologists but also by other health care practitioners with proper specific training [21], which may result in another advantage over CBT.

## Supporting information

S1 Appendix Description of the interventions. (DOCX)

S2 Appendix Complementary per-protocol analysis (Treatment vs no-treatment). (DOCX)

S3 Appendix Robustness analyses for CBT vs MI at 3 and 6 months. (DOCX)
