# Supplementary material for: Brief group-delivered motivational interviewing is equally effective as brief group-delivered cognitive-behavioral therapy at reducing alcohol use in risky college drinkers
Source: PLoS One. 2019 Dec 10;14(12):e0226271. doi: 10.1371/journal.pone.0226271 (PMC6903743; doi:10.1371/journal.pone.0226271)
Supplement: S1 Appendix — (DOCX) [file pone.0226271.s001.docx]

**Brief Cognitive Behavioral Therapy.** The first session began with an explanation of the general aim of the intervention; that is, to reduce or eliminate alcohol consumption beginning with that session. The second part was aimed at providing general information regarding the negative physical consequences of alcohol misuse. The third part was focused on: (i) developing basic skills to identify alcohol-related cognitions (i.e. beliefs, attitudes, and motives about alcohol consumption), and general automatic-irrational thoughts (e.g. overgeneralization, selective attention, arbitrary inference) as determinants of negative emotions; (ii) promoting reflection about how alcohol consumption increases negative emotions; and (iii) linking those pattern of thinking with problematic personal experiences related to alcohol consumption. The fourth part of the session was aimed at explaining behavioral techniques intended to prevent or reduce alcohol consumption (i.e. stimuli control and the development of alternative-incompatible behaviors). In the fifth part, participants were trained to complete a self-report about situations, thoughts, and feelings related to alcohol consumption experiences (i.e. their attempts to reduce or quit alcohol consumption). Participants were asked to fill out these self-reports during the week.

In the second session, following the psychoeducation component about the negative psychological consequences of alcohol use, participants were debriefed about the potential difficulties in fulfilling self-reports and their experiences maintaining abstinence or reducing alcohol consumption. Next, participants were trained in cognitive restructuring which centered on learning how to address general automatic-irrational thoughts by using a Socratic dialogue about their evidence, probability, and utility, and by promoting alternative ways of thinking regarding the effects of alcohol, and their attitudes and motives. The third part of this session was aimed at applying their learning to specific thoughts related to alcohol consumption experiences. The fourth part was focused on explaining specific cognitive and behavioral techniques to resist alcohol-related temptations (i.e. distraction), and maintaining potential reduction or abstinence (i.e. self-reinforcement and mastering). The self-report used in the second session included the identification of situations, thoughts, and feelings related to alcohol consumption experiences, as well as alternative thoughts used to overcome these experiences, and the feelings that those alternative thoughts produced.

The third session was focused on helping participants solve any problems that arose (i) during the fulfillment of self-reports, (ii) in their attempts to apply alternative rational thinking strategies, or (iii) when dealing with abstinence or when reducing alcohol consumption. Next, they were informed about the negative social consequences of alcohol misuse. The core component of this session involved training on preventing potential relapses. This was tackled by identifying common thoughts after a relapse (e.g., “it´s always going to be the same”; “I can’t do anything”), and by giving non-automatic alternatives and rational ways of thinking. Finally, the session was closed with a summary in which the psychologist highlighted the main information addressed during treatment.

**Brief Motivational Interviewing.** The psychologist used the following MI techniques during sessions: (i) open questions (to understand the patients’ internal world), (ii) reflective listening (to promote a deeper understanding of the meaning of the patients’ words, by means of an inferential statement), (iii) affirmations (to recognize patients´ worth, reflecting strengths, abilities, positive intentions, and past successes in achieving aims), and (iv) summaries (to compile information, suggest links between pieces of information with clinical value, and/or redirect the flow of the conversation through extended reflections). The dynamics of sessions were adapted to follow four different processes: (i) engaging, that is, the establishment of a therapeutic connection between the psychologist and participants, providing a propitious work environment; (ii) focusing, namely, the development of personal goals for the intervention; (iii) evoking, that is, eliciting the participants´ own motivation by reflecting their evolving dialogue (e.g., negative consequences of using alcohol that patients make explicit), and (iv) planning, that is, the preparation of a specific plan of action to change a behavior, (e.g. how to overcome the difficulties in achieving a goal). These processes were adapted in accordance with the participants’ stage of readiness to change (pre-contemplative or contemplative).

**The role of the psychologist.** In both interventions, the psychologist was responsible for creating and maintaining a therapeutic atmosphere of trust, respect, and warmth. These characteristics are common to most forms of therapy, regardless of their theoretical framework. In addition, the psychologist provided information about the goals of each therapy and explained homework assignments to participants, assessing their compliance and resolving any related questions. The psychologist also answered any doubts regarding the project, alcohol consumption, and/or any related issues raised by participants.

Apart from these shared characteristics, the psychologist played a different role in both interventions. In CBT, in addition to applying the aforementioned techniques, the psychologist was responsible for keeping a specific pace of work to order to include all the pre-established content. In this regard, he applied an active listening technique in which he gave participants the opportunity to express their thoughts at any time, and would kindly redirect the session to the planned content when necessary.

On the contrary, in the MI sessions, the psychologist played a more collaborative role through the use if open-ended questions and by encouraging all participants to express their feelings and thoughts. In addition, he employed a reflective listening technique in which he used inferential statements aimed at increasing the understanding of the patient´s words and creating thereby empathetic connections between the two in order to promote commitment and to facilitate the patient’s own motivation. It is worth noting that the psychologist also encouraged all participants to have a dynamic and participative role in the therapy. Simultaneously, the psychologist redirected the group with open questions following individual reflective statements in order to make sure that no participant would receive notably more attention than the others.
