# Supplementary material for: Brief group-delivered motivational interviewing is equally effective as brief group-delivered cognitive-behavioral therapy at reducing alcohol use in risky college drinkers
Source: PLoS One. 2019 Dec 10;14(12):e0226271. doi: 10.1371/journal.pone.0226271 (PMC6903743; doi:10.1371/journal.pone.0226271)
Supplement: S2 Appendix — (DOCX) [file pone.0226271.s002.docx]

In order to test the potential benefit of receiving one of the two treatments, we identified individuals who did not receive treatment in the two ITT groups (those who were intended to be treated, but ultimately were not), and those who received the intended treatment. Group (bMI, bCBT, No treatment) was recoded into two dummy variables. The first one (0, 1, 0) represents the potential advantage of bCBT compared to the other two conditions, whereas the second one (1, 0, 0) represents the potential advantage of receiving bMI compared to the other conditions.

The two dummy variables were included in the Bayesian regression analyses of TLFB change. Age was entered in the analysis as a continuous covariate due to the fact that it covaried with the observed reduction of alcohol consumption,. Two JZS Bayesian regressions with JASP defaults were run, one for the change in TLFB scores from baseline to the 3-month assessment, and the second for the change in TLFB scores from baseline to the 6-month assessment.

The first analysis (TLFB change, 3 months) yielded a Bayes Factors of 0.56 and 1.48 for the two dummy variables, respectively. The R^2^ was <0.001, 0.034, 0.001, and 0.046 for the null (including age), dummy 1, dummy 2, and both dummy variable models, respectively. The second analysis (TLFB change, 6 months) yielded Bayes Factors of 0.29 and 8.16 for dummy variables 1 and 2. The R^2^ was 0.162, 0.201, 0.307, and 0.307 for the null (including age), dummy 1, dummy 2, and both dummy variable models, respectively. In other words, at three months, treatment made no substantial difference with regard to TLFB change. At six months, however, the Bayes Factors showed substantial evidence supporting the null for bCBT, and supporting the alternative (namely, a substantial reduction of alcohol consumption for people who received the MI treatment). Positive effects were thus found only for MI at six months.
