# Supplementary material for: Brief group-delivered motivational interviewing is equally effective as brief group-delivered cognitive-behavioral therapy at reducing alcohol use in risky college drinkers
Source: PLoS One. 2019 Dec 10;14(12):e0226271. doi: 10.1371/journal.pone.0226271 (PMC6903743; doi:10.1371/journal.pone.0226271)
Supplement: S3 Appendix — (DOCX) [file pone.0226271.s003.docx]

**S3 Appendix. Robustness analyses for CBT vs MI at 3 and 6 months**


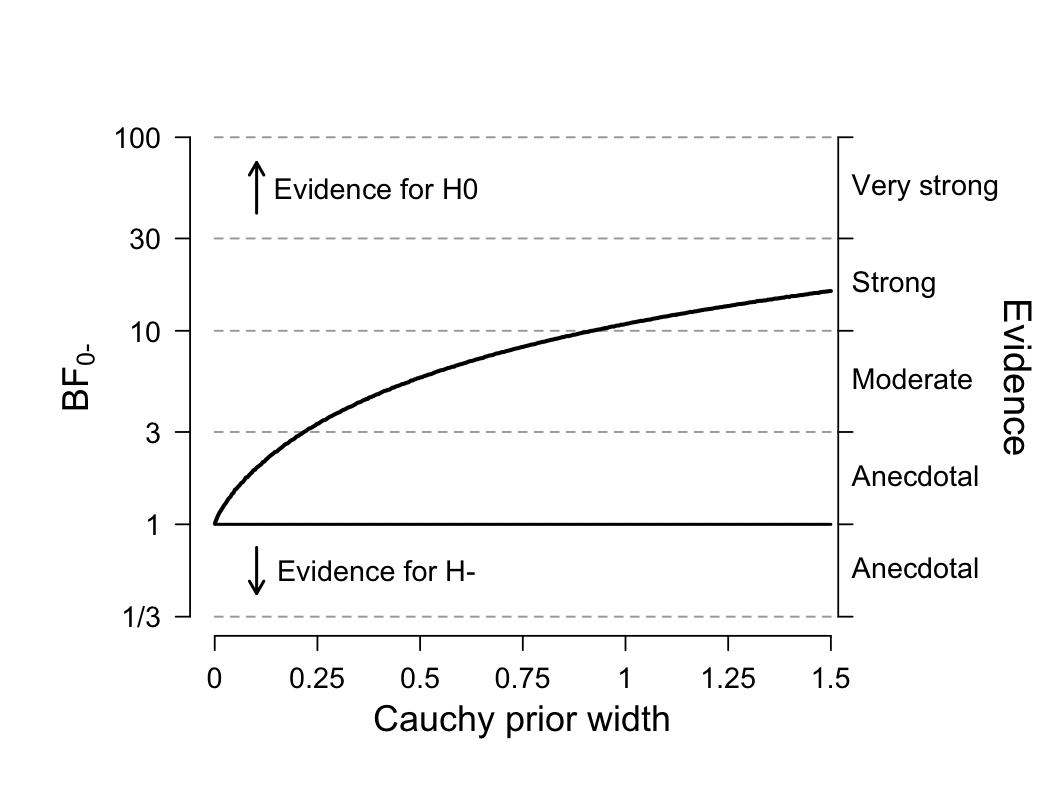


**S3.1 Fig. Robustness analysis for CBT vs MI at 3 months.**


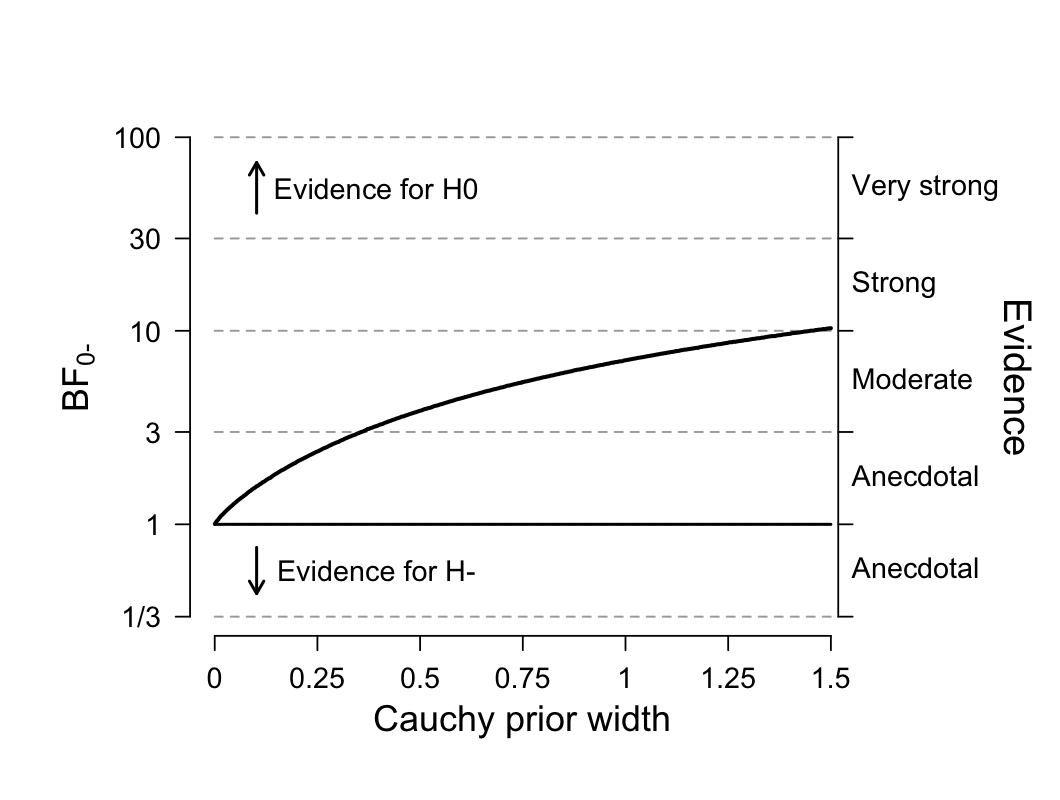


**S3.2 Fig. Robustness analysis for CBT vs MI at 6 months.**
